# Impact of the bidirectional relationship between communication and cognitive efficacy on orthopedic patient adherence behavior

**DOI:** 10.1186/s12913-022-07575-5

**Published:** 2022-02-14

**Authors:** Dong-Shang Chang, Wil-Lie Chen, Rouwen Wang

**Affiliations:** 1 Department of Business Administration, National Central University, Taoyuan, Taiwan; 2 School of Nursing, China Medical University, Taichung, Taiwan

**Keywords:** Bidirectional relationship, Quality of healthcare services, Physician-patient communication, Cognitive efficacy, Adherence behavior, Structural equation modeling

## Abstract

**Background:**

There is growing interest in patient autonomy, and communication between physicians and patients has become the essential cornerstone for improving the quality of healthcare services. Previous research has concentrated on the direct effect of physician-patient communication on service outcomes. In the present study, we examined the influence among constructs in the service process and the impact on healthcare outcomes. The present study used behavioral theory to expand the process aspect of the Donabedian healthcare service quality structure-process-outcome model to examine the impact of cognitive changes and communication feedback on patients’ adherence behavior. In addition, the moderating effect of hospital facility levels is examined.

**Methods:**

A conceptual model was developed and tested using a questionnaire administered to patients in eight hospitals. A total of 397 respondents returned usable surveys, with a response rate of 92.11%. Structural equation modeling was used to analyze the data in two steps that involved a measurement model and a structural model. The former was applied to estimate the Cronbach’s alphas, intercorrelations of factors, and descriptive statistics; the latter was used to test the hypothesized relationships of the constructs.

**Results:**

The results identified three mediators of the healthcare process within the healthcare services framework: physician-patient communication, cognitive efficacy, and adherence behavior. Physician-patient communication influenced cognitive efficacy (β = 0.16, *p* < 0.001), and cognitive efficacy influenced physician-patient communication (β = 0.18, *p* < 0.001). The effect of this bidirectional relationship on adherence behavior was positive (β = 0.38, *p* < 0.001). The healthcare structure influenced healthcare outcomes via these three healthcare process constructs. The adherence behavior of patients who were treated in the medical center has greater influences by the structure and physician-patient communication than it was treated in the regional hospitals.

**Conclusions:**

This study revealed a complex pattern in relationships among process constructs for healthcare services. The findings of this study acknowledge the important potential interrelationships among the healthcare service constructs to improve the quality of healthcare outcomes.

**Trial registration:**

CRREC104107. Date: 22/01/2016. Prospectively Registered.

**Supplementary Information:**

The online version contains supplementary material available at 10.1186/s12913-022-07575-5.

## Background

In the traditional paternalistic healthcare service decision-making model, the decisions related to health are almost dependent entirely on the physicians [1]. However, the traditional model is not sufficient for patient autonomy because with this model, it is difficult to enhance patients’ perceptions of healthcare quality and adherence behavior [1, 2]. Although most physicians independently make diagnostic decisions based on their professional knowledge, the lack of consensus with patients on treatment often leads to medical disputes in the treatment of chronic diseases [3]. A previous study demonstrated that medical teams with a low frequency of medical malpractice claims required physicians to be more communicative and exchange relevant information with their patients [4], such as providing risk information and paying attention to psychological needs. As the need to seek patients’ views and opinions is gradually being emphasized, shared decision-making has become a healthcare service trend [5]. In the context of shared decision-making, treatment decisions are derived from consensus of communication about patients’ preferences and efficacy knowledge rather than being based solely on their physical condition [5, 6]. The patients’ willingness to comply the physician’s orders is enhanced when patients and physicians exchange sufficient information [1, 7, 8]. These findings emphasize effective interactive processes are essential to ensure patients’ perceptions of healthcare service quality [6] and reduce the possibility of medical disputes [4].

Regarding the quality of healthcare services, the structure-process-outcome (SPO) model advocated by Donabedian [9, 10] has played a significant role in quality assurance and improvements in the healthcare field. This model has been used to evaluate the overall quality of healthcare in several areas, such as emergency general surgery [11], integrated chronic disease management [12], antenatal care services [13], lung cancer [14], and prostate cancer [15]. Although the Donabedian SPO model is the leading paradigm for evaluating the quality of healthcare services, it does not sufficiently recognize the complex and hidden interrelationships among healthcare service processes [16]. However, high quality and satisfaction of healthcare service often need patient involvement or compliance in the treatment process [17], which the provision of service process quality affects patients’ overall perception and choice. To encourage patient adherence behavior, the physicians must be addressed their intrapsychic factors [18], such as knowledge of the regimen, belief in the benefits of treatment, subjective norms, and attitudes toward medication-taking behavior. The empirical evaluation of psychological and behavioral treatment planning is a nascent area of study, and it is especially relevant in the treatment of chronic conditions [19, 20]. Correspondingly, Fishbein & Ajzen [21], first proposed the theory of reasoned action, which states strong relationship exists between one’s belief attitude and an individual’s conscious behavioral intentions. Several theoretical models have been developed to explain human acceptance and behavior intention, among which the theory of reasoned action (TRA) and technology acceptance model (TAM) are widely explored and extended frameworks for behavior intention studies [22]. The TRA is a theory that explains the general decision-making process of individuals’ behavior from the perspective of cognitive information and the value of expectations [21, 23]. Davis [24] proposed the TAM, adapted from the TRA, which contends that attitude comprises two core elements: perceived usefulness and perceived ease of use. The TAM theory has been modified by Venkatesh & Davis [25] and Venkatesh [26]. Finally, perceived usefulness and perceived ease of use (equivalent to performance expectancy and effort expectancy respectively) are regarded as critical determinants of behavior. In previous studies on patient participation in the decision-making process, scholars emphasized the necessity of medical-patient communication to improve service quality [5, 6]. Some studies have investigated the impact of medical-patient communication on the quality of medical services [4–6, 27, 28]; however, there are still insufficient studies on the correlation between cognitive belief and behavior of patients with chronic diseases.

Moreover, the concept of service quality is founded on the difference between personal cognitive expectations and perceived service outcomes [29, 30]. The quality of healthcare services is both an objective fact and a subjective judgment by patients [31, 32]. Because the quality of healthcare service is judged by different standards when different perspectives are considered, there is a cognitive gap of quality standards in understanding between physicians and patients [33, 34]. In short, healthcare service providers possess highly specialized medical knowledge, and few patients have equivalent knowledge or information [35]. Some previous studies have demonstrated that good communication between physicians and patients helped patients correctly understand their disease and increased their willingness to comply with treatment [36, 37], which improved the quality of healthcare services. Therefore, the process of consensus communication between physicians and patients is also an important consideration in medical services, which can lead to better medical outcomes [38]. Furthermore, Dibbelt et al. [39] state that recovery success of the intrapsychic and physical function was attributed to the healthcare process, including factors such as information exchange, expectation adjustment, and decision adherence behavior. Effective physician-patient communication is a basic part of the treatment process [5], which relieves the patient’s psychological burden and leads to better healthcare outcomes [11, 28, 32, 40]. Physicians and patients provide emotional support and exchange medical knowledge through communication [9, 41, 42]. However, efficacy is subjective and represents patients’ cognition of the degree of improvement in their physical condition after treatment [26, 43, 44]. Physicians must provide effective and thorough information and check the adequacy of patients’ understanding [27]. The sharing and communication of information have contributed to building a reality cognitive on successful treatment outcomes as well as hence the adherence behavior [5, 31].

For the reasons above, the purpose of this study was to use behavioral theory to expand the process aspect of the Donabedian SPO model of healthcare service quality and focus on examining the impact of physician-patient consensus on patient adherence behavior and healthcare outcomes. The framework of this study also focused on assessing the healthcare process, including physician-patient communication, cognitive efficacy, and adherence behavior.

## Methods

### Participants and procedures

A total of 431 questionnaires were distributed between September 2016 and January 2017, and 397 were usable for data analysis, for an overall response rate of 92.11%. The questionnaire was judged to be invalid if any items were missed or all the answers were selected as the same option. Before its general application, this survey was approved by the Research Ethics Committee (REC) of China Medical University and Hospital (CMUH).

This study investigated patients at the orthopedics departments of eight hospitals (five medical centers and three regional hospitals) in Taiwan. According to Taiwan’s hospital assessment, hospitals and medical facilities are divided into four levels, including medical centers, regional hospitals, district hospitals, and basic-level clinics. The type of hospital reflects its level of medical services and the number of beds and assures the representativeness of the sample in the country. The participants were sampled from the hospitals’ orthopedic healthcare service waiting for areas by using the convenience sampling method. Specifically, the participants were all over 20 years old, being treated with at least one orthopedic treatment at the representative hospitals, and provided informed consent to participate in the study. Three trained investigators distributed questionnaires face-to-face to the selected participants who were willing to complete them. The investigators proactively introduced themselves as members of the research team and spent an average of 15 min clearly informing the participants of relevant details before they filled the questionnaires, including data confidentiality, the participant’s rights, and the approximated time required to complete. The informed consent letter was given before the questionnaires were issued, and participants could withdraw if they felt uncomfortable.

### Measures

The questionnaire was divided into two parts: part 1 included demographic characteristics (e.g., sex, age, educational level); part 2 elicited constructs of orthopedic patients’ treatment experience including healthcare structure, physician-patient communication, cognitive efficacy, adherence behavior, and healthcare outcome. The questionnaire was developed based on the literature and adjusted to suit the healthcare setting in the Taiwanese context. Moreover, the items modified from previous interdisciplinary studies into the study context are necessary, because none of the structured instruments were specifically used to analysis the interacting process of orthopedic healthcare service before. The items used to capture the concept of healthcare structure and healthcare outcome were both adopted from Donabedian [9, 41, 42, 45]. The concept of communication between physicians and patients was adopted from Levinson et al. [4] and van Osch et al. [28]. The items used to capture the concept of the efficacy of patient cognition and adherence behavior were adopted from Venkatesh & Davis [25] and Venkatesh [26]. The succinct definition and their items of the adopted constructs were summarized in Table 1. All Items of the questionnaire are measured using a seven-point Likert scale (1 = strongly disagree; 7 = strongly agree). As such, the questionnaire of this study is appended accordingly (see Additional file 1).

**Table 1:** Operational definitions of the constructs

Construct	Definition	Item	Questions
Healthcare structure (HS)	This reflects the patient’s perceptions of the hospital context in which orthopedic care is provided.	HS1	Was the diagnosis and operation time provided by the physician adequate?
		HS2	Did the medical institution provide a comfortable treatment environment?
		HS3	Did the medical institution provide an undisturbed and private treatment environment?
		HS4	Was the service provided by the medical institution easy to obtain?
Physician-patient communication (PPC)	This reflects the patient’s perceptions of communication with physicians about treatment and care services.	PPC1	During the communication, did the physician care about your personal situation of daily activities?
		PPC2	During the communication, did the physician understand your anxiety?
		PPC3	During the communication, did the physician let you feel reliable?
		PPC4	During the communication, did the physician understand your concerns?
		PPC5	Did the physician praise you for following medical instructions?
Cognitive efficacy (CE)	This reflects the efficacy of the patient’s cognitive and expectations that the treatment would improve the physical condition.	CE1	Do you think the treatment can improve your physical state?
		CE2	Do you think the treatment can relieve your mental pain?
		CE3	Do you think the treatment can improve your daily activities?
Adherence behavior (AB)	This reflects the patient’s willingness and behavior intention of adherence to medical advice.	AB1	Did you follow the physician’s orders?
		AB2	Were you actively involved in decision-making about the treatment plan?
Healthcare outcome (HO)	This reflects the patient’s perceptions of the outcome of the treatment and the end result of improvement by orthopedic care.	HO1	Has your condition improved since the treatment?
		HO2	Has the mental pain caused by the disease been relieved since the treatment?
		HO3	Has your physical condition improved since the treatment?
		HO4	Have your daily activities improved since the treatment?

### Statistical analyses

In this study, structural equation modeling (SEM) in AMOS software version 21 was used to estimate the associations among latent constructs and observed variables in the concept model, as well as the degree to which a hypothesized model agrees with the observed data. Moreover, SEM also can explore the bidirectional relationship between constructs. When the relationship involves bidirectional paths, this pattern follows a non-recursive model. The non-recursive SEM assumes that the residuals are related or that there is reciprocal causation between the variables, which effectively solves complex real-life problems.

**Fig. 1:**
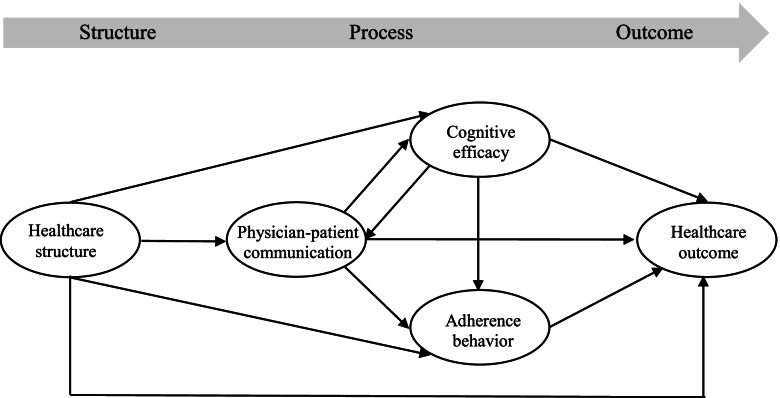
Conceptual model of the bidirectional relationship between process aspects in healthcare services

This study used behavioral theory to expand the process aspect of the Donabedian SPO model of healthcare service quality and focused on examining the impact of physician-patient consensus on patient adherence behavior and healthcare outcome. The Donabedian SPO model comprises three constructs: structure, process, and outcome [9], and the links between these three constructs have been widely confirmed in prior works [9, 45, 46]. Communication is an important construct that directly influences patients’ adherence intention [47], and indirectly influences behaviors via the patient’s perception of treatment efficacy. The adherence behavior of patients reflects their intention to comply with treatment plans [43]. Active interaction and communication between physicians and patients improve patient health and result in a positive treatment experience [39, 48]. The conceptual model of this study was illustrated in Fig. 1.

The model fit assessment was conducted after performing model specifications to evaluate how well a specified model fit the data. The threshold for acceptable goodness of fit is a normed chi-square (χ2/df < 5), goodness-of-fit index (GFI > 0.80), adjusted GFI (AGFI > 0.80), comparative fit index (CFI > 0.90), and root mean square error of approximation (RMSEA < 0.08) [49, 50]. No formal power calculation was done.

## Results

### Characteristics of samples

The respondents included 218 males (54.91%) and 179 females (45.09%). The demographic data revealed that 183 participants (46.10%) were between 20 and 29 years of age. Most respondents had college/university-level education (43.10%). Table 2 provides information on the characteristics of the respondents.

**Table 2:** Demographic characteristics of participants (*n* = 397)

Characteristics	n	%
Sex		
Male	218	54.91%
Female	179	45.09%
Age		
Between 20 and 29 years	183	46.10%
Between 30 and 39 years	56	14.10%
Between 40 and 49 years	30	7.60%
Between 50 and 59 years	40	10.10%
Older than 60 years	88	22.10%
Education level		
Less than High School	73	18.4%
High School	122	30.70%
College/University	171	43.10%
Postgraduate or above	31	7.80%

### Reliability and validity of the measured variables

Before performing factor causality testing, the examination results of this study provide support for the constructs of the conceptual model which are achieved validity and reliability (see Table 3). Specifically, this study follows the recommendations of Hair et al. [49] to assess the constructs of the conceptual model are achieved validity and reliability by examining convergent validity, internal consistency reliability, and discriminant validity. Convergent validity is evaluated by the standardized factor loadings (SFL) (λ) and average variance extracted (AVE), which assesses the average variance shared between the studied constructs and their individual items. As indicated in Table 3, the SFL values for all measurement items were greater than 0.7 [49], and the *t* values for them reached the level of significance (*p* < 0.001). Moreover, the AVE values of all constructs are well above the recommended 0.5 [49]. Thus, the measurement model demonstrated a satisfactory convergent validity. Furthermore, the internal consistency reliability of the instrument was measured using the composite reliability and Cronbach’s alpha. The internal consistency reliability describes whether the individual items all measure the same studied construct. Table 3 demonstrated that the values of internal consistency reliability measures are above 0.7 [49], as well indicated that our measures had fairly levels of reliability. Finally, the distinctiveness of the studied constructs in the measurement model was assessed by discriminant validity. The Fornell-Larcker (FL) [51] criterion was used to reveal whether the square root of the AVE in every latent variable is larger than the value of latent variable correlations (LVC). Table 3 reveals that the square root of AVE for all constructions is much larger than the corresponding LVC, and all studied constructs are assured to meet the discriminant validity. Overall, the results of the tests present that the proposed conceptual model in this study is reliable and valid.

**Table 3:** Results of the measurement model for the all constructs

Construct	Item	Convergent validity		Internal consistency reliability		Discriminant validity
		SFL^**a**^	AVE^**b**^	CR^**c**^	Cronbach’s alpha	FL criterion^**d**^
‘Rule of thumb’		> 0.7	> 0.5	> 0.7	> 0.7	Squared root of the AVE > LVC^e^
Healthcare structure (HS)	HS1	0.84^***^	0.75	0.92	0.92	Yes
	HS2	0.88^***^				
	HS3	0.89^***^				
	HS4	0.86^***^				
Physician-patient communication (PPC)	PPC1	0.83^***^	0.75	0.94	0.94	Yes
	PPC2	0.88^***^				
	PPC3	0.89^***^				
	PPC4	0.86^***^				
	PPC5	0.87^***^				
Cognitive efficacy (CE)	CE1	0.88^***^	0.82	0.93	0.93	Yes
	CE2	0.89^***^				
	CE3	0.93^***^				
Adherence behavior (AB)	AB1	0.85^***^	0.76	0.86	0.86	Yes
	AB2	0.89^***^				
Healthcare outcome (HO)	HO1	0.91^***^	0.81	0.94	0.94	Yes
	HO2	0.92^***^				
	HO3	0.92^***^				
	HO4	0.85^***^				

^***^p < 0.001

^a^*SFL* Standardized factor loadings, ^b^*AVE* Average variance extracted, ^c^*CR* Composite reliability, ^d^*FL criterion* Fornell and Larcker criterion, ^e^*LVC* Latent variable correlations

### The results of structural equation modeling

**Fig. 2:**
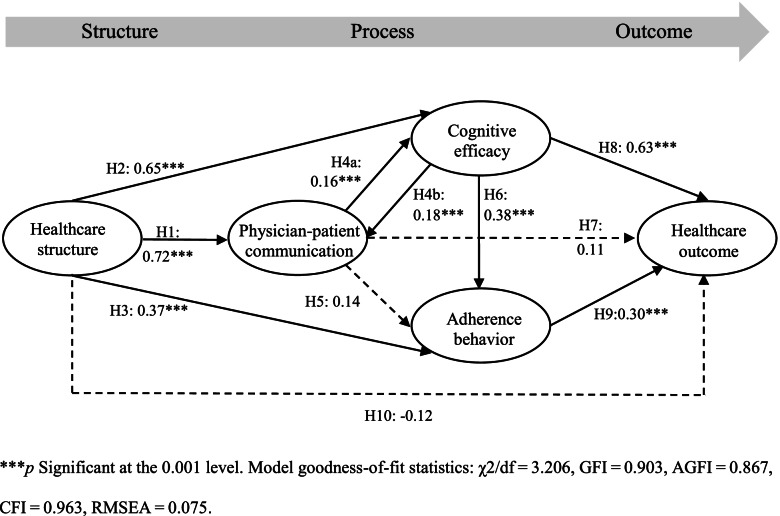
Empirical results of the bidirectional relationship between process aspects in healthcare services

The analysis results of the model fit show the proposed structural model is satisfactory (χ2/df = 3.206, GFI = 0.903, AGFI = 0.867, CFI = 0.963, and RMSEA = 0.075), and the hypothesized model corresponds with the observed data. Figure 2 shows the standardized estimates of the paths among the variables in the SEM. The healthcare structure directly influenced physician-patient communication (H1: *β* = 0.72, *p* < 0.001), cognitive efficacy (H2: *β* = 0.65, p < 0.001), and adherence behavior (H3: *β* = 0.37, *p* < 0.001). Regarding the healthcare process, there were correlations among the three mediators. Physician-patient communication influenced cognitive efficacy (H4a: *β* = 0.16, *p* < 0.001), and cognitive efficacy influenced physician-patient communication (H4b: *β* = 0.18, *p* < 0.001). This evidence indicates that the model is non-recursive, both paths differ significantly from 0, and the paths are almost equally strong in both directions. The relationship between physician-patient communication and patient cognitive efficacy formed a bidirectional loop. Physician-patient communication did not directly influence the patients’ adherence behavior (H5: *β* = 0.14, *p* = 0.12), but adherence behavior was directly influenced by the patients’ cognitive efficacy (H6: *β* = 0.38, *p* < 0.001). These test results could infer that physician-patient communication promoted patients’ adherence behavior through improvements in treatment efficacy cognition. Physician-patient communication did not directly influence the healthcare outcome (H7: *β* = 0.11, *p* = 0.17). The healthcare outcome was directly influenced by cognitive efficacy (H8: β = 0.63, *p* < 0.001) and adherence behavior (H9: β = 0.30, *p* < 0.001). From the perspective of overall healthcare services, the healthcare structure did not significantly influence the healthcare outcome (H10: β = − 0.12, *p* = 0.13). However, the healthcare structure indirectly influenced healthcare outcomes via the healthcare process.

### The multigroup analyses of SEM by hospital facility levels

A multigroup analysis of SEM was performed for the within-level factors with patients (lowest level units) nested within hospital facilities (upper-level units). We examined the moderating effect of hospital facility groups on the direct relationships. The sample is divided into two groups: those participants who were treated in the medical center (124 useable responses) and those participants who were treated in the regional hospital (273 useable responses). The *χ2* difference tests of multigroup moderation analysis revealed that the unconstrained model and restrained structural weights model were statistically different (see Table 4), *χ2* diff (22) = 55.42, *p* < .05, indicating that level of hospital facility moderated one or more structural parameters in the model.

**Table 4:** Multi-group analysis with hospital facility levels as a moderator

Models	***χ***^**2**^***(df)***	***χ***^**2**^***/df***	***χ***^**2**^(df)	RMSEA	CFI
Model with no restrictions	633.56(251)	2.52	–	0.06	0.95
Model with restricted structural weights	688.98(273)	2.52	55.42(22)***	0.06	0.94

^***^p < 0.001, *df* Degrees of freedom, *RMSEA* Root mean square error of approximation, *CFI* Comparative fit index

To determine whether the relationship parameters among the constructs of healthcare services were significantly different between medical centers and regional hospitals, each path in turn was constrained to be equal across groups. The results demonstrated that the path between healthcare structure and physician-patient communication is significantly different between medical centers and regional hospitals. Specifically, the effect of structure on communication was greater for medical centers (B = 1.03) than it was for regional hospitals (B = 0.72). Moreover, the influences between healthcare structure, physician-patient communication, and cognitive efficacy on adherence behavior are significantly different between medical centers and regional hospitals. The effect of structure on adherence behavior was stronger for medical centers (B = 1.03) than it was for the regional hospitals (B = 0.25). The effect of physician-patient communication on adherence behavior was greater for medical centers (B = − 0.46) than it was for the regional hospitals (B = 0.20). The effect of cognitive efficacy on adherence behavior was stronger for the regional hospitals (B = 0.53) than it was for medical centers (B = 0.18). The results of multigroup analyses by hospital facility levels on the relationship among the constructs of healthcare service were summarized in Table 5.

**Table 5:** Multigroup moderation analysis of hospital facility levels on the relationships of healthcare service (*N* = 397)

Hypothesis		Medical center (***N*** = 124)		Regional hospital (***N*** = 273)	
		Estimate^**a**^	***p***-value	Estimate^**a**^	***p***-value
H_m1_	HS➔PPC	1.03	0.00	0.72	0.00
H_m2_	HS➔CE	0.78	0.00	0.78	0.00
H_m3_	HS➔AB	1.03	0.01	0.25	0.03
H_m4_	HS➔HO	0.12	0.84	−0.15	0.11
H_m5_	PPC➔CE	0.17	0.00	0.17	0.00
H_m6_	PPC➔AB	−0.46	0.13	0.20	0.05
H_m7_	PPC➔HO	−0.09	0.82	0.15	0.05
H_m8_	CE➔PPC	0.17	0.00	0.17	0.00
H_m9_	CE➔AB	0.18	0.05	0.53	0.00
H_m10_	CE➔HO	0.74	0.00	0.60	0.00
H_m11_	AB➔HO	0.26	0.36	0.31	0.00

^a^*Estimate* Unstandardized coefficient, *HS* Healthcare structure, *PPC* Physician-patient communication, *CE* Cognitive efficacy, *AB* Adherence behavior, *HO* Healthcare outcome

## Discussion

Our analytical findings are supported by previous study results that confirmed a significant link between healthcare structure and healthcare outcomes and found that the healthcare process played an important role in the relationship between these two constructs. Therefore, our research further examined the relationships among constructs in the healthcare process to help explain the impact of changes in patients’ perceptions and behavior on the perceived quality of healthcare services.

With increased autonomy, healthcare service providers have begun to accept and encourage patient participation in the healthcare service process and joint decision-making about treatments. The purpose of such patient involvement is to allow physicians to more clearly identify patients’ expectations regarding treatment outcomes and to allow patients to express their preferences during treatment. Butkus et al. [52] demonstrated that negative emotions or a lack of patient engagement in the context of communication with physicians adversely affected efficacy and was associated with increased patient anger and frustration, deterioration of recall, and increased healthcare malpractice claims. If a patient fails to understand the side effects of medications or early treatment cessation, then good healthcare outcomes cannot be obtained. A study of patients with adult spinal deformity showed that the average postoperative recall was only 18% after 6 to 8 weeks [53]. Conversely, when the patient had a good emotional experience during interaction with the physician, their psychological burden was reduced, and better healthcare outcomes were achieved [32]. The results of the present study correspondently verified the correlation between the latent constructs in the healthcare process and their impact on the quality of healthcare services.

We found a bidirectional relationship between physician-patient communication and patient cognitive efficacy, which influenced patient adherence behavior. The continuous feedback of this bidirectional relationship was similar to the process of communication and interaction. Good communication makes the patient feel that they are involved, and they are more willing to comply with a jointly determined treatment schedule [54]. This finding is supported by shared decision-making models in which clinicians provide information about treatment options and listen to patients’ preferences and cognitive values to ensure that both parties are involved in decision-making [54, 55]. Physician-patient communication and perceived efficiency are decisive constructs for the success of shared medical decision-making in the healthcare process. The cooperation of both parties reduces the occurrence of medical disputes and provides high-quality healthcare services that are satisfactory to both parties. Moreover, the results of this study also found that the adherence behavior of patients who were treated in the medical center has a greater influence by the structure and physician-patient communication than it was treated in the regional hospitals. The impact of structure on communication was greater for medical centers than it was for regional hospitals.

The findings of this study are of theoretical and practical significance, but there are some research limitations. First, this study was limited to a single specialty, and thus our findings are most likely a representation of the situation in orthopedic healthcare departments. This specialty was selected for several reasons. The incidence rate of knee joint injuries has increased rapidly in recent years, partially due to an increase in jogging [56], and with the aging of the population, there has been an increase in the incidence of osteoarthritis (OA), which is a progressive and incurable joint disease [57]. Second, the use of convenience sampling could be viewed as a limitation of the study due to potential selection bias. Therefore, the sampling strategy may make the findings here lack generalizability, but it is still a crucial study using the SEM approach to analyze the relationships between the physician-patient consensus on patient adherence behavior as well as expand the process aspect of the Donabedian SPO model, which could impact future research in this area. Third, the study lacks a power calculation with respect to the number of visit patients. The lack of power calculation may have decreased the possibility of detecting statistically significant. However, the findings of the study suggest that the model still offers reliable and useful information by meeting the rule of minimum sample size, model-fit indexes, and the statistically significant *p*-value even if the power analysis has not been performed. Although the findings may not be representative of the entire country, this study aimed to include appropriate and sufficient representative data to estimate the hypothesized model under time and budget limitations, as well as to inform power calculations for a future trial.

## Conclusions

This study empirically tested theories of healthcare service quality in terms of the perceptions and behavior of orthopedic patients. This study expanded on Donabedian’s SPO model and behavioral decision theory, and our hypotheses were supported. Specifically, this study revealed the potential relationship between the three constructs of the healthcare process: physical-patient communication, cognitive efficacy, and adherence behavior. The healthcare structure significantly influenced the healthcare outcome via these three constructs. These findings highlight communication, patient understanding, and patient behavior as important constructs of healthcare service processes and demonstrate their potential beneficial effects on healthcare outcomes. This study presented the results of empirical tests, and its findings may aid the development of new applications and future studies on the related topics of healthcare quality and shared decision-making.

## Supplementary Information

**Additional file 1.** Questionnaire developed for this study.

## Data Availability

The data set used and analyzed during the current study are available from the corresponding author on reasonable request.

## Abbreviations

SEM: Structural equation modeling
HS: Healthcare structure
HP: Healthcare process
PPC: Physician-patient communication
CE: Cognitive efficacy
AB: Adherence behavior
HO: Healthcare outcome
SFL: Standard factor loading
AVE: Average variance extracted
LVC: Latent variable correlations
CR: Composite reliability
DV: Discriminant validity
GFI: Goodness-of-fit index
AGFI: Adjusted goodness-of-fit index
CFI: Comparative fit index
RMSEA: Root mean square error of approximation
OA: Osteoarthritis
