# Supplementary material for: Impact of the bidirectional relationship between communication and cognitive efficacy on orthopedic patient adherence behavior
Source: BMC Health Serv Res. 2022 Feb 14;22:199. doi: 10.1186/s12913-022-07575-5 (PMC8845262; doi:10.1186/s12913-022-07575-5)
Supplement: Supplementary file 1 — Additional file 1. Questionnaire developed for this study. [file 12913_2022_7575_MOESM1_ESM.docx]

Additional file 1. Questionnaire developed for this study.

*Dear Participant,*

*We invite you to participate in this brief (15-minute) survey to know your opinion about some aspects of the healthcare that you have received as a patient with orthopedic diseases.*

*By completing this survey, you are consenting to participate in this study; however, your participation in this survey is voluntary. You may choose not to participate. If you decide to participate in this survey, you may withdraw at any time. Your participation and/or withdrawal from this study will have no impact on your relationship with the researchers or their affiliated institutions. You also have the right to ask any questions about the study at any time. The survey is anonymous and the authors will not collect any data that could enable the identification of the participants.*

*We encourage you to participate since your opinion will be very useful to have real data on the opinion of patients, which can be used to improve healthcare service for patients with orthopedic diseases.*

*We really appreciate your willingness to participate and value your feedback, and wish you all the best. For more information on this study please contact Mrs. Rouwen Wang at 103481023@cc.ncu.edu.tw. Dr.* *Dong-Shang Chang (changds@mgt.ncu.edu.tw) and Dr. Wil-Lie Chen (wlchen7108@gmail.com) are the study PI and Co-PI.*

*Thank you,*

*Dong-Shang Chang, Wil-Lie Chen, and Rouwen Wang*

*The following survey is divided into two parts. In the first one, we request your opinion about the treatment experience with orthopedic diseases. In the second part, we request your demographic information.*

| PART A: Treatment experience of orthopedic patients | | | | | | | | |
| --- | --- | --- | --- | --- | --- | --- | --- | --- |
| Please rate how much you experienced on each item below as; the scale ranges from 1, for “strongly disagree” to 7, for “strongly agree.” | | | | | | | | |
| 1. Was the diagnosis and operation time provided by the physician adequate? | | | | | | | | |
|  | 1 | 2 | 3 | 4 | 5 | 6 | 7 |  |
|  | Disagree Strongly | Disagree | Disagree Somewhat | Neutral | Agree Somewhat | Agree | Agree Strongly |  |
|  |  |  |  |  |  |  |  |  |
| 2. Did the medical institution provide a comfortable treatment environment? | | | | | | | | |
|  | 1 | 2 | 3 | 4 | 5 | 6 | 7 |  |
|  | Disagree Strongly | Disagree | Disagree Somewhat | Neutral | Agree Somewhat | Agree | Agree Strongly |  |
|  |  |  |  |  |  |  |  |  |
| 3. Did the medical institution provide an undisturbed and private treatment environment? | | | | | | | | |
|  | 1 | 2 | 3 | 4 | 5 | 6 | 7 |  |
|  | Disagree Strongly | Disagree | Disagree Somewhat | Neutral | Agree Somewhat | Agree | Agree Strongly |  |
|  |  |  |  |  |  |  |  |  |
| 4. Was the service provided by the medical institution easy to obtain? | | | | | | | | |
|  | 1 | 2 | 3 | 4 | 5 | 6 | 7 |  |
|  | Disagree Strongly | Disagree | Disagree Somewhat | Neutral | Agree Somewhat | Agree | Agree Strongly |  |
|  |  |  |  |  |  |  |  |  |
| 5. During the communication, did the physician care about your personal situation of daily activities? | | | | | | | | |
|  | 1 | 2 | 3 | 4 | 5 | 6 | 7 |  |
|  | Disagree Strongly | Disagree | Disagree Somewhat | Neutral | Agree Somewhat | Agree | Agree Strongly |  |
|  |  |  |  |  |  |  |  |  |

*-- Survey continues on the back of the page--*

| 6. During the communication, did the physician understand your anxiety? | | | | | | | | |
| --- | --- | --- | --- | --- | --- | --- | --- | --- |
|  | 1 | 2 | 3 | 4 | 5 | 6 | 7 |  |
|  | Disagree Strongly | Disagree | Disagree Somewhat | Neutral | Agree Somewhat | Agree | Agree Strongly |  |
|  |  |  |  |  |  |  |  |  |
| 7. During the communication, did the physician let you feel reliable? | | | | | | | | |
|  | 1 | 2 | 3 | 4 | 5 | 6 | 7 |  |
|  | Disagree Strongly | Disagree | Disagree Somewhat | Neutral | Agree Somewhat | Agree | Agree Strongly |  |
|  |  |  |  |  |  |  |  |  |
| 8. During the communication, did the physician understand your concerns? | | | | | | | | |
|  | 1 | 2 | 3 | 4 | 5 | 6 | 7 |  |
|  | Disagree Strongly | Disagree | Disagree Somewhat | Neutral | Agree Somewhat | Agree | Agree Strongly |  |
|  |  |  |  |  |  |  |  |  |
| 9. Did the physician praise you for following medical instructions? | | | | | | | | |
|  | 1 | 2 | 3 | 4 | 5 | 6 | 7 |  |
|  | Disagree Strongly | Disagree | Disagree Somewhat | Neutral | Agree Somewhat | Agree | Agree Strongly |  |
|  |  |  |  |  |  |  |  |  |
| 10. Do you think the treatment can improve your physical state? | | | | | | | | |
|  | 1 | 2 | 3 | 4 | 5 | 6 | 7 |  |
|  | Disagree Strongly | Disagree | Disagree Somewhat | Neutral | Agree Somewhat | Agree | Agree Strongly |  |
|  |  |  |  |  |  |  |  |  |
| 11. Do you think the treatment can relieve your mental pain? | | | | | | | | |
|  | 1 | 2 | 3 | 4 | 5 | 6 | 7 |  |
|  | Disagree Strongly | Disagree | Disagree Somewhat | Neutral | Agree Somewhat | Agree | Agree Strongly |  |
|  |  |  |  |  |  |  |  |  |
| 12. Do you think the treatment can improve your daily activities? | | | | | | | | |
|  | 1 | 2 | 3 | 4 | 5 | 6 | 7 |  |
|  | Disagree Strongly | Disagree | Disagree Somewhat | Neutral | Agree Somewhat | Agree | Agree Strongly |  |
|  |  |  |  |  |  |  |  |  |

*-- Survey continues on the back of the page--*

| 13. Did you follow the physician’s orders? | | | | | | | | |
| --- | --- | --- | --- | --- | --- | --- | --- | --- |
|  | 1 | 2 | 3 | 4 | 5 | 6 | 7 |  |
|  | Disagree Strongly | Disagree | Disagree Somewhat | Neutral | Agree Somewhat | Agree | Agree Strongly |  |
|  |  |  |  |  |  |  |  |  |
| 14. Were you actively involved in decision-making about the treatment plan? | | | | | | | | |
|  | 1 | 2 | 3 | 4 | 5 | 6 | 7 |  |
|  | Disagree Strongly | Disagree | Disagree Somewhat | Neutral | Agree Somewhat | Agree | Agree Strongly |  |
|  |  |  |  |  |  |  |  |  |
| 15. Has your condition improved since the treatment? | | | | | | | | |
|  | 1 | 2 | 3 | 4 | 5 | 6 | 7 |  |
|  | Disagree Strongly | Disagree | Disagree Somewhat | Neutral | Agree Somewhat | Agree | Agree Strongly |  |
|  |  |  |  |  |  |  |  |  |
| 16. Has the mental pain caused by the disease been relieved since the treatment? | | | | | | | | |
|  | 1 | 2 | 3 | 4 | 5 | 6 | 7 |  |
|  | Disagree Strongly | Disagree | Disagree Somewhat | Neutral | Agree Somewhat | Agree | Agree Strongly |  |
|  |  |  |  |  |  |  |  |  |
| 17. Has your physical condition improved since the treatment? | | | | | | | | |
|  | 1 | 2 | 3 | 4 | 5 | 6 | 7 |  |
|  | Disagree Strongly | Disagree | Disagree Somewhat | Neutral | Agree Somewhat | Agree | Agree Strongly |  |
|  |  |  |  |  |  |  |  |  |
| 18. Have your daily activities improved since the treatment? | | | | | | | | |
|  | 1 | 2 | 3 | 4 | 5 | 6 | 7 |  |
|  | Disagree Strongly | Disagree | Disagree Somewhat | Neutral | Agree Somewhat | Agree | Agree Strongly |  |
|  |  |  |  |  |  |  |  |  |

*-- Survey continues on the back of the page--*

| **PART B: Demographics** | | |
| --- | --- | --- |
| **1. What is your sex?** | | |
|  |  | Male |
|  |  | Female |
| **2. Which category below includes your age?** | | |
|  |  | Between 20 and 29 years |
|  |  | Between 30 and 39 years |
|  |  | Between 40 and 49 years |
|  |  | Between 50 and 59 years |
|  |  | Older than 60 years |
| **3. What is your highest qualification?** | | |
|  |  | Less than High School |
|  |  | High School |
|  |  | College/University |
|  |  | Postgraduate or above |

*Thank you very much for completing this survey.*
